# Interactions between the omnivorous bug *Nesidiocoris tenuis* (Heteroptera: Miridae) and the tomato pests *Helicoverpa armigera* (Lepidoptera: Noctuidae) and *Phthorimaea absoluta* (Lepidoptera: Gelechiidae): predation, phytophagy, and prey preference

**DOI:** 10.1093/jisesa/iead056

**Published:** 2023-07-10

**Authors:** Félicien Abègnonhou Totin, Delano Ronald Togbé, Antonio Sinzogan, Miriam Frida Karlsson

**Affiliations:** International Institute of Tropical Agriculture (IITA), 08 BP 0932-Tri Postal, Cotonou, Benin; Department of Crop Production, Faculty of Agronomic Sciences (FSA), University of Abomey-Calavi (UAC), 03 BP 2819 Cotonou, Benin; International Institute of Tropical Agriculture (IITA), 08 BP 0932-Tri Postal, Cotonou, Benin; Department of Crop Production, Faculty of Agronomic Sciences (FSA), University of Abomey-Calavi (UAC), 03 BP 2819 Cotonou, Benin; International Institute of Tropical Agriculture (IITA), 08 BP 0932-Tri Postal, Cotonou, Benin; Department of Ecology, Swedish University of Agricultural Sciences (SLU), SE-750 07 Uppsala, Sweden

**Keywords:** biological control, zoophytophagous, preference, functional response

## Abstract

*Nesidiocoris tenuis* (Reuter) (Heteroptera: Miridae) is a zoophytophagous bug that can derive nutrients from 3 trophic levels: plants, herbivorous arthropods, and other predators. On tomato, besides damaging the plants as they feed, might the mirid also forage on pest species and repel pests. In greenhouse and laboratory experiments, we investigated the functional response of the bug, its prey preference, and its influence on the oviposition potentials of 2 major pest species *Helicoverpa armigera* (Hübner) (Lepidoptera: Noctuidae) and *Phthorimaea absoluta* Meyrick (Lepidoptera: Gelechiidae) on tomato *Solanum lycopersicum* L. (Solanaceae). *Nesidiocoris tenuis* showed a Type II functional response to both prey species. The estimated handling time was higher for *H. armigera* eggs than for *P. absoluta* yet *N. tenuis* attack rates did not differ between the 2 prey species. *Nesidiocoris tenuis* did not show a preference for 1 species when prey eggs were provided in equal proportions. The feeding on tomato plants by *N. tenuis* did not affect oviposition by the 2 moth species, as neither species showed a preference for clean or *N. tenuis-*adult-damaged plants and clean or *N. tenuis-*nymph-damaged plants. This study shows that *N. tenuis* can prey upon eggs of both moth species as the 3 species co-occur in tomato fields. However, because of the shorter handling time of *P. absoluta* eggs by the predator and the higher number of eggs laid by *H. armigera*, the co-occurrence might be less detrimental to the *H. armigera* populations compared to *P. absoluta.*

## Introduction

Zoophytophagous predators provide pest control on plants but can also affect plant growth as plant-feeders in periods of prey scarcity. One such species is *Nesidiocoris tenuis* (Reuter) (Heteroptera: Miridae), an omnivorous bug species used for the biological control of several pest species (Pérez-Hedo and Urbaneja 2016). It belongs to a large group of generalists mirid predators used to manage tomato pests (Urbaneja et al. 2012, Pérez-Hedo and Urbaneja 2014). *Nesidiocoris tenuis* is distributed worldwide yet temperatures between 20 and 35 °C are suitable for population growth and multiplication of the bug (Gavkare et al. 2021). The third, fourth, and fifth nymphal instars of the mirid bug consume more prey per individual than the adults or the first and second nymphal instars (Mollá et al. 2013). Due to its entomophagy on eggs, small larvae, and nymphs of several insects species, *N. tenuis* can contribute to the control of whiteflies, thrips, leafhoppers, leaf miners, spider mites, aphids, and moths species, including the invasive tomato leafminer *Phthorimaea absoluta* Meyrick (Lepidoptera: Gelechiidae) (Solsoloy et al. 1994, Calvo et al. 2012, Gavkare and Sharma 2016, Ferracini et al. 2019, Pazyuk 2020), and other lepidopteran such as *Helicoverpa armigera* (Hübner) (Lepidoptera: Noctuidae) (Komala Devi et al. 2002), and *Spodoptera littura* Fabricius (Lepidoptera: Noctuidae) (Wei et al. 1998). However, the mirid bug can also feed on plants by inserting its stylet in the plant cells (Chinchilla-Ramírez et al. 2021). This plant feeding results in necrotic rings that appear on the stems, leaves, and petioles of the plants, and causes flower abortion and drop, thereby leading to poor fruit set and fruit malformations (Arnó et al. 2010), particularly when arthropod prey is scare (Castañé et al. 2011). This reduction of fruit number however can be compensated by an increased weight of the remaining fruits (Sánchez and Lacasa 2008). While feeding on the plants, the zoophytophagous predator concomitantly activates or up-regulates both abscisic acid (ABA) and jasmonic acid (JA), volatile compounds that act as defence signals against certain herbivorous species making those plants less attractive to pests (Pérez-Hedo et al. 2015b). The repellency of some insects by the plant (Pérez-Hedo et al. 2015a, 2017) results in reduced feeding, reduced oviposition, and reduced fecundity in those phytophagous insects (Ataide et al. 2016, Pérez-Hedo et al. 2018, Zhang et al. 2018b). Both herbivores and their natural enemies can be affected by the presence of the mirid bug *N. tenuis* on the plants (Pérez-Hedo et al. 2015b, Pappas et al. 2017, Zhang et al. 2018a). Because of its various interactions with the plant, both as a predator and as phytophagous species, using the mirid bug as a biological control agent in plant protection has been debated (Sánchez et al. 2006, Sánchez 2008, Pérez-Hedo and Urbaneja 2016, Puentes et al. 2018, Siscaro et al. 2019).

Insect pests against which *N. tenuis* can be used as biological control agent include the fruit borer *H. armigera* and the tomato leafminer *P. absoluta*, 2 of the most devastating pests of tomato in West Africa (Diatte et al. 2016, 2018). *Helicoverpa armigera* is highly polyphagous and has a wide geographical distribution (Reed and Pawar 1982). The caterpillars feed on leaves, stems, buds, inflorescences, and fruits of more than 200 plants, including cotton, and several solanaceous species such as sweet pepper, chilli pepper, eggplant, tobacco, and tomato (CABI 2022). Tomato fruit loss in West Africa due to *H. armigera* can reach up to 42% (Mailafiya et al. 2014, Diatte et al. 2018). *Phthorimeae absoluta*, it is an invasive insect pest in Europe, Asia, and Africa (OEPP/EPPO 2005), and has been considered the most damaging pest on tomato in Latin America for decades (Guedes and Picanço 2012). Outbreaks of this pest were recently reported in several west African countries (Pfeiffer et al. 2013, Son et al. 2017, Karlsson et al. 2018, Fiaboe et al. 2020), where it is capable of causing yield losses of up to 80–100% in absence of appropriate control methods (Desneux et al. 2010). Unfortunately, these 2 pests (*H. armigera* and *P. absoluta*) can occur simultaneously on tomato plants, in several parts of the world resulting in tremendous losses (Tropea Garzia et al. 2012, Diatte et al. 2018). One of the management strategies thought to control these pests is the use of their natural enemies. For that purpose, one of most common natural enemies used in greenhouses in Europe is the predatory bug *N. tenuis* (Komala Devi et al. 2002, Romeis and Shanower 2010, Dehliz and Guénaoui 2015), and this mirid is also found on tomato plants in West Africa (Garba et al. 2020, Kouadio et al. 2022). However, how this zoophytophagous predator interacts with the 2 lepidopteran pest species on tomato plants is still under-explored. One of the key parameters for measuring the efficacy of a predator is its functional response, a description of changes in prey consumption by the predator in response to prey density. It helps to predict the speed of prey density decrease in relation to a specific predator density (Enkegaard et al. 2001, Kalyebi et al. 2005, Pervez and Omkar 2005, Abraços-Duarte et al. 2021). The functional response of a predator depends on prey species (Hassell et al. 1977), prey size (Streams 1994), predator age and satiation status (Eveleigh and Chant 1982), and on environmental conditions (Audenaert et al. 2014). To understand the functioning of these multitrophic interactions among the predatory bug *N. tenuis* and the 2 lepidopteran pest species (*H. armigera* and *P. absoluta*) laboratory and greenhouse studies were conducted. We evaluated if *N. tenuis* shows preference for any of the pest species and we determined the functional response pattern of the predator to both pest species. In addition, how phytophagy by the predatory bug interferes with the oviposition preference of the 2 moth species on tomato plants was investigated.

## Materials and Methods

### Plant Material

Tomato, *S. lycopersicum* var. Padma (EastWest Seed), was used for insect species mass-rearing and laboratory trials. The variety Padma is known to be resistant to bacterial wilt (*Ralstonia solanacearum*) widespread in Benin (Sikirou et al. 2017). Tomato seeds were initially sown individually in plastic trays. After 3 weeks, seedlings were transferred into plastic pots (13 cm in diameter) and maintained in a greenhouse at 27 ± 3 °C, RH 88 ± 8%, and a photoperiod of 12L:12D. Pesticide-free tomato plants, fertilized with compost, were used for the experiments at approximatively 1 month of age.

### Insect Material

The predatory bug *N*. *tenuis* individuals were collected from tomato fields in southern Benin near the towns Abomey-Calavi, Grand-Popo, Allada, and Ouidah. They were mass-reared in the rearing facilities at the International Institute of Topical Agriculture (IITA-Benin). The *N. tenuis* rearing was initiated from a colony of about 100 adults and 50 nymphs, released in a Plexiglass cage (50 × 50 × 50 cm) hosting potted tomato plants. *Nesidiocoris tenuis* were provided ad libitum with fall armyworm *Spodoptera frugiperda* (J. E. Smith) (Lepidoptera: Noctuidae) egg masses (laid on tissue paper). Eggs were obtained through a rearing of *S. frugiperda* that were fed on corn under laboratory conditions and the initial rearing started from specimens collected in corn in southern Benin. Eggs for food were renewed every 2 days as they could hatch after 2–3 days. To obtain experimental individuals of *N. tenuis*, first or second instar nymphs were regularly transferred from rearing cages and placed in separate cages (15 × 15 × 15 cm), provided with *S. frugiperda* eggs, tomato leaflets, and water. They were observed every day until they developed to fourth instar or adult according to the stage needed. All rearing cages were kept at 25 ± 2 °C, RH 85 ± 5%, and a photoperiod of 12L:12D.

The mass-rearing of *H. armigera* was initiated from larvae collected from tomato fields located in southern Benin, near the towns of Ouidah and Allada. Larvae were reared on an artificial diet consisting of a mixture of corn flour (30%), cowpea flour (45%), honey (5%), yeast (10%), ascorbic acid (2%), sorbic acid (1%), methyl-p-hydroxybenzoate (1%), agar (5%), formaldehyde (1%), and tap water, following the methodology described by Ahmed et al. (1998). The diet was renewed every 2 days until pupation. After pupation, pupae were collected and isolated in plastic boxes (17 cm in diameter) until adult emergence. Young adults (5 days old) were used for the experiments.

The *P. absoluta* individuals used in the experiments were initially collected from tomato fields in Ouidah, and from tomato production plots installed at IITA-Benin station. Newly emerged *P. absoluta* were transferred into a new Plexiglass cage (50 × 50 × 50 cm) containing 3–4 potted tomato plants that were used for oviposition. After every 2 days, a new potted tomato plant was provided and the old infested plants were removed and kept in other cages and regularly provided with fresh plants until adult emergence. Three-day-old *P. absoluta* adults were used for the experiments.

### Effect of *N. tenuis* Damage on Oviposition by *H. armigera* and *P. absoluta* on Tomato

A two-choice assay was conducted in a greenhouse (27 ± 3 °C, RH 88 ± 8%, 12L:12D) to assess the oviposition behavior of *H. armigera* and *P. absoluta* when offered simultaneously intact (nondamaged) and *N. tenuis*-damaged (punctured) tomato plants. A prospecting study of Miridae species was carried out in the field and nymphs and adults were counted on tomato plants, we found 8 ± 2 *Nesidiocoris* spp. individuals per plant (unpublished data). Therefore, we used 10 *N. tenuis* individuals per plant to obtain damaged plants in this study. We exposed potted tomato plants in Plexiglass cages (50 × 50 × 50 cm) to either 10 adults or 10 fourth instar *N. tenuis* nymphs for 24 h. The predators were then removed from the cages using an aspirator. Adult moths were then introduced into 1 cage with 1 damaged and 1 nondamaged tomato plant. Adult moths were allowed to mate for 24 h in a separate cage (15 × 15 × 15 cm) prior to the experiment, so only mating couples were used. Either 1 couple (♂+♀) of *H. armigera* or 5 couples of *P. absoluta* as *H. armigera* female lays 5–6 times more eggs than *P. absoluta*. The moths were left in the cage and allowed to oviposit on the tomato plants for 24 h after which the number of eggs laid on each plant were counted using an Optivisor magnifying glass. The experiment was conducted over 2 months and replicated 20 times per moth species and predator stage (adult and nymph) with approximately 4 cages per species tested simultaneously.

### Prey Preference by *N. tenuis* between *H. armigera* and *P. absoluta* Eggs

In a laboratory experiment (25 ± 2 °C, RH 85 ± 5%, 12L:12D), *N. tenuis* nymphs were simultaneously offered eggs of *H. armigera* and eggs of *P. absoluta* to determine the preference of the predator. Each moth species was first allowed to infest fresh tomato plants for 24 h to obtain the eggs. Infested leaves were removed from the tomato plants and leaflets harboring at least 10 eggs were selected. Additional eggs were removed with a camel hair brush. Two leaflets containing 10 eggs of either moth species were placed in a Petri dish (8.5 cm in diameter) on top of moistened cotton wool to maintain humidity and 1 fourth instar nymph of *N. tenuis* was deposited in each dish. The predator was then allowed to feed and after 24 h, the number of unconsumed eggs was recorded. This experiment was replicated 40 times.

### Functional Response of *N. tenuis* to Eggs of *H. armigera* or *P. absoluta*

To determine how prey consumption by *N. tenuis* varied with prey density, we conducted a functional-response assay under greenhouse conditions (27 ± 3 °C, RH 88 ± 8%, 12L:12D). To obtain prey eggs, 1 tomato plant was offered to either 1 couple (♂+♀) of *H. armigera* or 5 couples (♂+♀) of *P. absoluta* in different Plexiglass cages (50 × 50 × 50 cm). The plants were removed from the cages after 24 h and the number of eggs laid was counted and recorded using an Optivisor magnifying glass. As eggs of both moths are very fragile, we did not handle them after being laid on the plants, as they could be damaged. Thereafter, each plant was transferred to another Plexiglass cage and 1 fourth-instar nymph of *N. tenuis* was deposited on the plant using a camel hair brush and allowed to feed on the eggs laid on the plants for 24 h. Thereafter, the plants were removed from the cages and the number of eggs remaining after consumption by the predator was counted and recorded. Prey replacement was not done in this experiment. An individual predator was never tested more than once. The difference between the number of eggs initially laid by the moths and the number remaining after consumption by the predators gave us the exact number of eggs consumed. As initial densities of eggs laid by the pests on the plants were highly variable, classes were formed, and the central values (10, 40, 70, 190) of each class are used as fixed densities for the data analysis (Table 1). Predators that did not consume eggs during the experiment were removed from the data before statistical analysis. Thus, we had 34 replicates for *H. armigera* and 38 replicates for *P. absoluta* for data analysis.

**Table 1.** Paired-wise comparison of number (mean ± SE) of *P. absoluta* and *H. armigera* eggs eaten by *N. tenuis* when given different densities of the eggs

Densities	H. armigera		P. absoluta	
	Replicates	Mean (SE)	Replicates	Mean (SE)
10	12	5.17 (0.843)b	12	5.92 (0.927)b
40	6	10.33 (1.990)ab	21	13.48 (1.315)a
70	4	9.25 (2.237)ab	3	25.33 (5.947)a
190	12	15.25 (1.926)a	2	21.00 (6.185)a

Means (SE) in a row followed by different letters are significantly different.

### Statistical Analyses

The analysis was performed using R version 3.5.3 statistical software package (R Core Team 2019). A paired t-test analysis was performed to determine the impact of the predator’s previous presence (i.e., damage on tomato plants) on the oviposition by the 2 moth species. *Nesidiocoris tenuis* prey preference was determined using the β-Manly preference index developed by Manly (1974) and calculated as follows:


$$\begin{array}{l} \beta_{i}=\frac{log\left(\frac{r_{i}}{Ri}\right)}{\overset{m}{\underset{j=1}{\sum }}\left(log\left(\frac{r_{j}}{R_{j}}\right)\right)} \end{array}$$

With $\beta_{i}$ representing the predator’s preference for prey i, $r_{i}$, and $r_{j}$ the numbers of prey i and j not eaten. $R_{i}$ and $R_{j}$ are the initial numbers of the prey species and m the number of prey type classes. The β values were calculated for each replicate and averaged to determine the mean β value. If β is close to 1, the predator prefers prey i, and if it is close to 0, prey j is preferred. An index value close to 0.5 indicates no preference. We took into account the depletion of prey by the predator during the experiment. Means of β preference index for each prey species were compared using Student’s t-tests.

An ANOVA was performed followed by a pair-wise comparison of the mean consumption at the different densities to determine significant differences. The effect of prey density on the predator consumption was tested by using the Generalized Linear Models with negative binomial distribution. Functional response model developed by Rogers (1972) was then used to describe how *N. tenuis* consumption changed with availability of *P. absoluta* and *H. armigera* eggs. The model assumes the depletion of eggs and the number of eggs eaten $N_{e}$ was modeled through equation (1) (Pritchard et al. 2017) for which the solution equation (2), was obtained by using Lambert-W function described by Corless et al. (1996).


$$\begin{array}{l} N_{e}=N_{0}\left(1-exp\left(aN_{0}^{q}\left(hN_{e}-T\right)\right)\right) \end{array}$$ (1)

$$\begin{array}{l} N_{e}=N_{0}-\frac{W\left(ahN_{0}^{1+q}exp\left(aN_{0}^{q}\left(hN_{0}-T\right)\right)\right)}{aN_{0}^{q}h} \end{array}$$ (2)

In these equations, $N_{0}$ is the initial density of eggs; T the experimental time (24 h); a the instantaneous eggs attack rate of *N. tenuis* per unit of time; h the handling time (in hours): the time spent subjugating, ingesting, and digesting each egg item and q a scaling exponent defining the extent to which the functional response change from type II (q=0) to type III (q>0).

The analysis was performed using the package *FRAIR*, version 0.5.100 (Pritchard et al. 2017). The frair_test() function which uses logistic regression of the proportion of eggs eaten as function of the initial density $N_{0}$ was run to determine the shape or type of the functional response by determining if the data fit a type II or III functional response, given each prey species (*P. absoluta*, *H. armigera*). After determining the type of functional response, frair_fit() function was used to estimate the attack rate (*a*) and the handling time h. frair_compare() was then used to test whether there are differences between the parameters earlier estimated throughout frair_fit(). This comparison assumes that there are no differences between fitted parameters.

## Results

### Effect of *N. tenuis* Damage on Oviposition by *H. armigera* and *P. absoluta* on Tomato Plant

No significant difference in number of laid eggs on damaged and nondamaged tomato plants was observed, neither by *P. absoluta* or *H. armigera* nor when damaged by *N. tenuis* adults or nymphs (Table 2).

**Table 2.** *Helicoverpa armigera* and *P. absoluta* oviposition on nondamaged versus *N. tenuis*-punctured tomato plants

Moth species	*N. tenuis* stage	Mean no. eggs (±SE)		*t*	df	*P*
		Intact	Punctured plants			
*H. armigera*	Adult	57.95 (14.20)	82.45 (19.81)	1.3334	19	0.1982
*H. armigera*	Nymph	43.8 (14.63)	47.65 (18.47)	0.25726	19	0.7997
*P. absoluta*	Adult	41.45 (5.72)	30.65 (4.88)	−1.5048	19	0.1488
*P. absoluta*	Nymph	24.75 (4.04)	19.6 (4.07)	0.91354	19	0.3724

### Prey Preference by *N. tenuis* between *H. armigera* and *P. absoluta* Eggs

*Nesidiocoris tenuis* consumed 5.95 ± 0.47 eggs of *P. absoluta* and 6.72 ± 0.5 eggs of *H. armigera* per day. The consumption of the 2 prey species indicated no significant preference for either species, giving Manly’s β index of 0.49 ± 0.04 for *P. absoluta* and 0.51 ± 0.04 for *H. armigera*. Comparison of preference indices using *t*-test also indicated no significant predator preference of the predator between prey species (*t* = −0.22048, df = 39, *P* = 0.8266).

### Functional Response of *N. tenuis* to *H. armigera* and *P. absoluta* Eggs

**Fig. 1.**
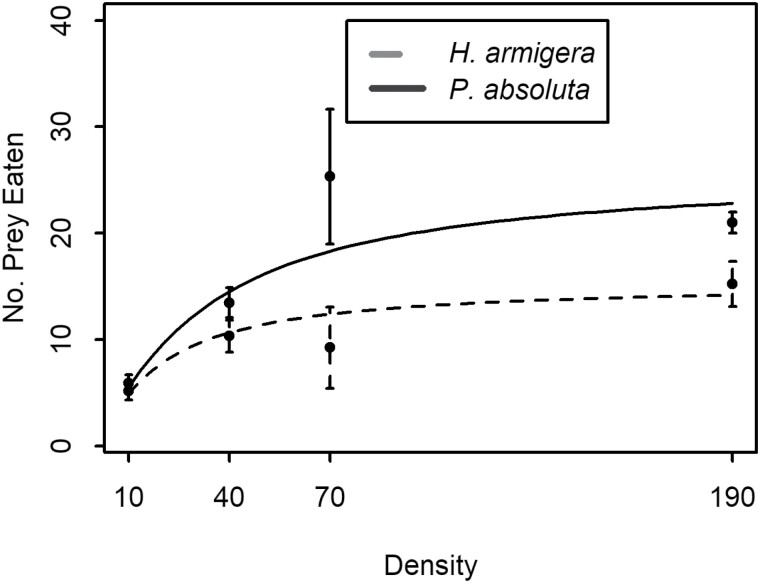
Type II functional response curve for number of eggs (means ± SE) of *H. armigera* (dash line) and *P. absoluta* (full line) attacked in 24 h by *N. tenuis* as a function of egg densities.

*Nesidiocoris tenuis* overall daily consumption did not differ between the 2 prey species (*F* = 3.064; df = 1; *P* = 0.0849). The functional response was a type II, regardless of the type of prey considered (Table 3). Irrespective of prey species, the rate of prey consumption by *N. tenuis* increased with prey density before leveling-off to reach a plateau. However, the level of prey consumption was higher for *P. absoluta* than for *H. armigera* (Fig. 1). The estimated handling time was higher for *H. armigera* (1.562 h^–1^) than for *P. absoluta* (0.921 h^–1^) while the attack rates did not differ for the 2 prey species (Da=0.0;P=0.977) (Table 3). The daily maximum number of eggs that could be eaten by *N. tenuis* was estimated at 26 *P. absoluta* eggs and 15 *H. armigera* eggs.

**Table 3.** Type of functional response for *N. tenuis* on its prey *P. absoluta* and *H. armigera* and the mean estimated values (±SE) of attack rate (a), handling time (h), and maximum number of eggs that could be attacked (T/h) and result of difference in attack rate (Da) and handling times (Dh) between the 2 species

Prey	Type	*a*	*h*	*T*/*h*	D*a*	D*h*
*P. absoluta* and *H. armigera*	II	0.050 (0.006)***	0.130 (0.074)***	185	**–**	**–**
*P. absoluta*	II	0.042 (0.007)***	0.921 (0.117)***	26	<0.0001 (0.010)NS	−0.641 (0.169)***
*H. armigera*	II	0.042 (0.008)***	1.561 (0.122)***	15		

*P < 0.05, **P < 0.01, ***P < 0.001, NS = nonsignificant.

## Discussion

This study is of key importance in clarifying the role that the zoophytophagous *N. tenuis* may play in the biological control of *H. armigera* and *P. absoluta*, 2 devastating pests of tomato. Our study revealed that *N. tenuis* did not show preference for *P. absoluta* over *H. armigera* eggs or vice versa when the 2 moth species were presented together. Our results also suggest that in a tomato field *N. tenuis* would feed eggs of both prey species as they encounter them. However, *P. absoluta* eggs which are mainly singly laid on the underside of tomato leaves could be more exposed, since this part of the tomato plant is where *N. tenuis* nymphs are usually present, while *H. armigera* lay single eggs more often on top of the leaves. Furthermore, because of the shorter handling time of *P. absoluta* eggs by the predator and the higher number of eggs laid by *H. armigera*, the co-occurrence of *N. tenuis* might be less detrimental to the *H. armigera* populations compared to *P. absoluta.*

Oviposition by either of the moth species was not affected by previous feeding on a tomato plant by *N. tenuis* nymphs or adults. Behavioral shifts in the target pest might be a nonlethal effect that a biological control agent can cause, adding to the direct lethal effect of biological control (Culshaw-Maurer et al. 2020). Plant feeding by *N. tenuis* adults and nymphs may cause the plant to release volatile compounds, caused through an upregulation of JA and ABA genes (Naselli et al. 2016). These volatiles emitted by the plant have been shown to repel the whitefly *Bemisia tabaci* (Gennadius) (Hemiptera: Aleyrodidae) and attract its parasitoid *Encarsia formosa* Gahan (Hymeoptera: Aphelinidae) (Naselli et al. 2016). In laboratory experiments, the spider mite *Tetranychus urticae* Kock (Acari: Tetranychidae) did not respond to odors from tomato plants fed upon by *N. tenuis* in Y olfactometer tubes and oviposited the same number of eggs on intact and fed upon tomato plants (Pérez-Hedo et al. 2018). However, tomato plants fed upon by *N. tenuis* resulted in antixenosis behavior by *P. absoluta* (Pérez-Hedo et al. 2015a, 2015b). Nevertheless, we did not observe that previous presence of the predator *N. tenuis* affected the moth oviposition behavior on tomato plants, as there was no difference in the number of eggs laid on nondamaged and *N. tenuis*-punctured tomato plants. This disparity might be due to the number of predators used for puncturing the tomato plant, as we used 10 predators per plant while Pérez-Hedo et al. (2015b) introduced 100 *N. tenuis* to 4 tomato plants for 24 h to induce emission of behaviorally active volatiles. The number of predators might have been insufficient to trigger enough volatile cues that could have deterred the moths to oviposit on the damaged plants. Indeed, Pappas et al. (2015) observed that the amount of herbivore oviposition was dependent upon the density of the predator to which the plant had been exposed. The lack of an ovipositional response by either moth species in this study may also be due to the variety of the tomato plant used in the studies. The quantity and composition of the volatiles emitted may vary from species to species and among genotypes of the same species (Loughrin et al. 1995, Hoballah et al. 2002).

The predator *N. tenuis* exhibited a type II functional response when feeding on eggs of both moth species. Previous functional response assessments have revealed both type II and III functional response by *N. tenuis*. Ziaei Madbouni et al. (2017) observed a type II functional response while testing in temperatures between 15 and 30 °C but a type III at 35 °C. Sharifian et al. (2015) observed however that *N. tenuis* exhibited a type II functional response when feeding on *P. absoluta* and Ephestia *kuehniella* at 25 °C while Michaelides et al. (2018), a III functional response when feeding on *P. absoluta* eggs at 25 °C. Differences between function II and III have to do with predation results at low prey densities, and might be related to the environment where eggs were placed and to factors that affect the predator preference. Our estimation of *N. tenuis* maximum daily egg consumption was 34 and 27 for *P. absoluta* and *H. armigera* respectively, which was slightly lower than previously estimated to approximately 50 eggs/day (Michaelides et al. 2018). Functional response results indicate that *N. tenuis* is able to feed on both pest species in tomato fields and are able to switch prey in case of shortage of one of them. However, as *N. tenuis* has a low reproductive rate compared to both pests, conservation of and/or releases in the target ecosystems will be required. This will help to enhance its density in tomato fields.

It emerged from our results that the handling time of *P. absoluta* eggs by the predatory bug was significantly shorter than that for *H. armigera* eggs. It appears then that it is easier for *N. tenuis* fifth-instar nymph to predate on eggs of *P. absoluta* than on those of *H. armigera*. In fact, the estimated handling time includes time spent not only on actual prey handling but also on other nonsearching activities (Hassell 1978). The handling time estimate is the cumulative effect of time taken during capturing, killing, subduing, and digesting the prey (Veeravel and Baskaran, 1997). The longer time spent by the predator handling *H. armigera* eggs could be because they became sated sooner due to the larger size of the *H. armigera* eggs compared to *P. absoluta* eggs (Queiroz-Santos et al. 2018, OEPP/EPPO 2005). Handling time may be proportional to the size of the prey item (Streams 1994, Aljetlawi et al. 2004).

Biological control with *N. tenuis* is a controversial issue yet does its predation of numerous pest species justify the use and its importance as a biological control agent in crop production (Pérez-Hedo and Urbaneja 2016). Our results indicate that *P. absoluta* will be more negatively affected than *H. armigera*. The decline of *P. absoluta* infestations in tomato fields in recent years in sub-Saharan Africa, particularly in Benin could be partly explained by this efficiency of the predator on this pest. However, it would be interesting to carry out more trials in semicontrolled or open field conditions to better understand the behavior of the predator in conditions where the moths are not confined or forced to lay eggs on a single plant. This would be of particular interest as both pests occur at the same time, that is, at the beginning of the tomato production season.
